# Traditional Chinese Medicine for Pediatric Allergic Diseases

**DOI:** 10.1007/s11882-025-01208-7

**Published:** 2025-07-31

**Authors:** Michelle Carnazza, Robert Werner, Morgan Begley, Nan Yang, Raj Tiwari, Jan Geliebter, Xiu-Min Li

**Affiliations:** 1 General Nutraceutical Technology LLC, Elmsford, NY 10523 USA; 2 Department of Pathology, Microbiology & Immunology, New York Medical College, Valhalla, NY 10595 USA; 3 Department of Otolaryngology, New York Medical College, Valhalla, NY 10595 USA; 4 Department of Dermatology, New York Medical College, Valhalla, NY 10595 USA

**Keywords:** Traditional chinese medicine, Pediatric allergy, Food allergy, Eosinophilic esophagitis, Eczema, Allergic rhinitis, Allergic asthma

## Abstract

**Purpose of Review:**

There is a clinically important and unmet need for long-term safe and effective preventative and therapeutic options for pediatric allergic diseases. This communication reviewed the use of Traditional Chinese Medicine (TCM) in pediatric allergic disease, including eczema, urticaria, eosinophilic esophagitis (EoE), food allergy (FA), asthma, and allergic rhinitis.

**Recent Findings:**

Through evaluation of case studies, series, or clinical trials of pediatric allergy patients, or in vitro studies involving samples collected from pediatric allergy patients and in vivo model systems, naturally occurring small molecule compounds’ mechanism of action by evidence-based scientific outcomes were elucidated. Notable clinical outcomes include reduction in severity score, reduction across various allergic diseases that demonstrated no toxicity, no severe adverse effects, and are well-tolerated. Immunological outcomes that attribute to this include a switch from Th2-mediated allergic response to a Th1/Treg response, characterized by reduced total and specific IgE, total eosinophil counts, and levels of exotoxin, TNF-a, IL-6, IL-8, IL-5, and IL-4, with elevated levels of IFN-γ and IL-10. For eczema, both multiple and single herbal formulations are common treatment modalities, including internal administration and external herbal baths and creams, whereby both provide substantial beneficial outcomes. For EoE, internally administrated formulations and use of acupuncture have been reported and shown mitigation of allergic responses. In FA, formulations have been studied in clinical trials showing consistent safety, with protection remaining to be established. More advanced development of single herbal compounds may provide an advantage for use in FA treatment. For allergic rhinitis, several multiple herbal formulations and acupuncture have demonstrated improved symptom scores. Similarly, in asthma herbal formulas and acupuncture were highly clinically effective.

**Summary:**

TCM has demonstrated high safety and efficacy in both preclinical and clinical models of various allergic diseases, including eczema, food allergy, eosinophilic esophagitis, allergic rhinitis, and asthma. Therefore, this scientific evidence suggests that naturally occurring small molecule compounds are promising preventives and therapeutics for pediatric allergic diseases.

**Disclosure:**

All reported studies/experiments with human or animal subjects performed by the authors have been previously published and complied with all applicable ethical standards (including the Helsinki declaration and its amendments, institutional/national research committee standards, and national/institutional guidelines).

## Introduction

There has been an increased prevalence of allergic conditions in children over the last five decades. This “allergy epidemic” in the developed world is thought to be the result of the inflammatory western diet, improved hygiene promoting an uneducated immune system, epigenetic activation and silencing of critical genes, and the allergen march [1]. While allergy is a non-communicable inflammatory disease, natural progression of allergic disease has been demonstrated [2]. There is an 85% probability of development of food allergies, allergic asthma, or environmental allergies when having eczema [3]. The two major hypotheses for this include the damaged skin barrier brought about by inflammation, allowing for absorption of allergens, and the shared genetic and environmental risk factors [3]. Therefore, suggesting that with the onset of eczema, there is an opportunity for the development of other allergic diseases.

The pathology of allergic disease is centralized around IgE. When IgE coats and is crosslinked on a mast cell or basophil surface, this causes IgE-mediated inflammation through the release of histamine, leukotrienes, and Th2 cytokines IL-4 and IL-13 from its granules [4]. The site where the body confronts the allergen and releases these inflammatory mediators, largely histamine, are responsible for the manifestation of symptoms. In western medicine, symptom management is at the forefront, as there are no conventional cures to combat the allergic response [5]. This includes avoidance of triggers, using corticosteroids and epinephrine, and immunotherapy. Avoidance is ineffective and propagates anxiety around the child and their family. While rescue medications and immunotherapies are effective, there are some therapeutic limitations, especially with long-term use. For example, steroids begin to irritate the skin and disturb the immune system, and antihistamines are very potent and lose their effect after a few days. For immunotherapy the dose must be increased gradually, causing more adverse effects, and with cessation of treatment, sensitization is restored. Unfortunately, with that, children with allergic conditions see an increase in healthcare use and cost with a decreased quality of life and potentially life-threatening outcomes [6]. Therefore, there is a dire, unmet need for the identification of novel preventative and therapeutic options in allergic disease.

Traditional Chinese medicine (TCM) is an attractive therapeutic strategy, as generally they are effective, low cost, and produce fewer side effects. This medical system utilizes herbs in the form of lotions, creams, baths, teas, and pills and sometimes acupuncture, and acupressure. Naturally occurring compounds have demonstrated immunomodulatory properties and hence the potential to be utilized as therapeutic options in diseases like cancer, diabetes, and asthma. Traditional and Chinese herbal medicine compounds have been reported to modulate allergic responses, serving as both preventative and treatment options. Clinical trials, case studies, primary articles, as well as abstracts and unpublished data have been investigated here for their use in pediatric allergic diseases.

## Pediatric Eczema (Atopic Dermatitis) and Hives (Urticaria)

### Pediatric Eczema

Eczema (atopic dermatitis) is a condition of great global priority, as prevalence and morbidity are on the rise. Studies have demonstrated that eczema burden is substantial, at about 6% of children and adolescents globally. The lifetime prevalence of eczema is about 15% to 30% in children and 2% to 10% in adults. Studies have demonstrated that eczema burden is substantial, at as many as 30% of children and adolescents [7–9] and 10.8% of children in the United States [9]. Chronic eczema impairs quality of life, both physically and emotionally. Additionally, there are psychosocial problems and a financial burden on families. Therefore, there is a need for reliable, long-term effect of treatments for eczema, as topical corticosteroid “addition” or withdrawal syndrome (Named TSA/TSW) can occur. This “steroid addiction” exacerbates the difficulty in symptom management, including erythroderma and papulopustular development, severe sleep disturbance, and frequent Staphylococcus infection. Current biologics that target the Th2 immune response can be effective in some patients but entail high cost, hypersensitivity reactions including anaphylaxis, and indefinite treatment [10, 11] therefore, there is a need for safer, alternative eczema treatments for children.

### Single and Multi-Herbal Formulas for Pediatric Eczema

TCM has been well-documented as eczema treatment, including herbs and acupuncture (Table 1). Analysis of case studies in Taiwan demonstrated that the most common form of TCM for children with eczema was herbal medicine, with the most common single herbs being *Glycyrrhiza uralensis*, *Dictamnus dasycarpus*, and *Cryptotympana pustulata* and the most common herbal formula being Xiao-Feng-San (XFS) [12]. Further, it was evident that usage of TCM is typically higher in older children, likely due to poor control of the eczema when younger and dissatisfaction with the western treatment options [12]. Analysis of 8 clinical trials demonstrated that treatment with Chinese herbal medicine improved lesion severity, lesion size, and sleep score (*p* = 0.01) [13]. When comparing adverse effects, Chinese herbal medicine is deemed to be safe [13]. This was validated in another meta-analysis of 17 clinical studies, whereby Chinese herbal medicine correlated with a higher overall recovery rate and decreased recurrence following cessation of therapy [14].

**Table 1:** Summary of TCM effects on pediatric eczema, urticaria, eoe, and food allergy

Treatment	Clinical Trials/ Human Cells	Mechanism of Action	Ref
*Pediatric Eczema*			
**Medicated bath of TCM combined with tacrolimus**	Average age: 6 years old Group A (*n* = 34) 0.05% desonide ointment Group B (n-34) 0.03% tacrolimus ointment Group C (*n* = 34) medicated both of TCM and tacrolimus Lasted until 1 week after skin lesion elimination or for 3 weeks and follow up 3 months EASI scores decreased Incidence of adverse reaction decreased Recurrence rate decreased	Serum LTB_4_ and LTC_4_ and DLQI scores were all decreased successively	[16]
**Combined TCM** *Phellodendron chinesis* herbal bath additive, herbal cream (*Fructus tribuli*,* Radicis angelicae sinensis*,* Flos lonicerae*,* Potentillae chinensis Herba*, and *Indigo naturalis*), and internal tea (*Sclerotium Poriae Cocos*,* Cortex moutan*,* Fructus kochiae*,* Massa fermentata praeparata*,* Flos Lonicerae*,* Radix arnebiae*,* Fructus forsythiae*,* Indigo naturalis*,* Radix glycyrrhizae*)	Tolerated well by all patients, including those with steroid withdrawal syndrome Favorable response of patients with moderate to severe atopic dermatitis Disease severity scores decreased Normal liver and kidney function	Reduction in Th2-mediated allergic response Total serum IgE level decreased Peanut-specific IgE decreased Absolute eosinophil counts decreased	[17, 18]
**Group A (PTQXT)**: Radix Pseudostellariae *Forsythia suspensa*,* Ramulus Uncariae cum Uncis*,* Medulla Junci*,* Herba Lophatheri*,* Semen Coicis*,* Rhizoma Dioscoreae*,* Concha Ostreae*,* Radix Glycyrrhizae* **Group B (PTQXT + External Wash)** External wash: *Flos Lonicerae*,* Rhizoma Polygonati*,* Herba Menthae*,* Radix Glycyrrhizae*	Mean SCORAD decreased gradually Significantly greater improvement in quality of life No severe adverse events		[19]
**External herbal bath**,** cream**,** and oral tea**	Sample size: *n* = 28 Received TCM at least 3 months Cohort 1: *n* = 10, ages 6–48 months, topical steroids with inadequate response Cohort 2: *n* = 8, ages 2–40 years, worsening eczema associated with sudden topical steroid withdraw Cohort 3: *n* = 10, ages 1–13 years, high total IgE Reduced SCORAD, steroid use, and reduced sleep disturbance	Elevated blood eosinophils in Clinical Trial Decreased skin eosinophil counts in vivo mouse models Dose-dependent reduction of IgE, TNF-alpha, and exotoxin production in vitro	[21]
*Pediatric Urticaria*			
**Remedy A-D** **Herbal bath additive**: *Phellodendron chinesis* formula: *Cortex Phellodendri*,* Radix Rhizoma Rhei*,* Radix Sophorae Flavescentis*,* Cortex Dictamni*,* Dayscarpi Radicis*,* Fructus Tribuli Terrestris*,* and Rhizoma Smilacis Glabrae* **Herbal cream III**: *Phellodendron chinesis* topical cream (2.8%) with 1% *Indigo naturalis* extracts **Shi Zhen Tea 1a**: *Herba Schizonepetae*,* Cicada Molting*,* Bombyx Batryticatus*,* Fructus Arctii Lappae*,* Rhizoma Atractylodis*,* Macrocephalae*,* Sophora favescences Ait* **Mei Huang Tea**: *Pruni Mume* formula: *Prunus mume*,* Zanthoxylum schinifolium*,* Angelica sinesis*,* Zingiber officinalis*,* Cinnamomum cassia*,* Phellodendron chinesis*,* Panax giseng*,* Ganoderma lucidum*	Combined decrease in incidence of atopic events: hive episodes, epinephrine doses, diphenhydramine doses, prednisone doses, allergy-related emergency room visits	Total and food-specific IgE reduced	[30]
*Pediatric Eosinophilic Esophagitis*			
**Digestive Tea Formula**: 7,4 dihydroxy flavone (DHF) of *Glycyrrhiza uralensis*	Remained symptom-free for 6 months but with rare events intensity and duration were reduced Complete remission of EoE with normal tissue and biopsy of 0–7 eosinophils/ hpf	Inhibition of Eotaxin, Th2 cytokines (TNF-alpha, IL-6, IL-8, and IL1-beta) and IgE DHF can directly bind TNF- α	[39–41]
Pediatric Food Allergy			
**FAHF-2**	Acute Phase I- 7 days (Median: 16 years; Range: 12–33) No grade 3 adverse events Extended Phase I- 6 months (Median: 16 years; Range: 12–27) Phase II- 6 months (Median: 16 years; Range: 12–45)	Reduced IL-5 and increased IFN-Y and IL-10 Reduction in CD63 + basophil activation Increased CD4 + CD25 + FoxP3 + Treg cells	[59–62]
**E-B-FAHF-2**: Combination trial of Omalizumab, multi-OIT, and E-B-FAHF-2 or Placebo	Phase II- 2 years of treatment (Median: 11.7 years; Range: 6–40) Follow up for remission status	Decreased sIgE of both EOIT and OIT groups Decreased milk SPT in EOIT compared to OIT	[62]
**Arctigenin**	PBMCs from 26 food-allergic patients	Reduced IgE + B cells upon anti-CD40/IL-4 stimulation No effect on IgG1 or IgG4 Upregulation of Bcl6 Downregulation of B cell proliferation and survival genes (UBE2C, CCNB1, BIRC5, and MKI67) Reduced IL-5 and IL-13	[63]

*Abbreviations*: EASI (Eczema Area and Severity Index), DLQI (Dermatology Life Quality Index), LT (leukotriene) SCORAD (Scoring of Atopic Dermatitis), TNF (Tumor Necrosis Factor), PBMC (peripheral blood mononuclear cells), OIT (oral immunotherapy), SPT (skin prick test)

### Herbal Bath Therapy for Pediatric Eczema

Chinese herbal bath therapy has demonstrated effectiveness in the treatment of eczema in children in China [15]. In theory, herbal bath therapy works through penetration of the drugs with the warm effect into the skin, meridian points, and blood vessels [15]. The herbal solutions are thought to be absorbed by the sweat glands, mucous membranes, and capillaries to produce both local and systemic effects, while also affecting the nerve ending receptors in the skin that regulate nerves, body fluid, and circulation [15]. A meta-analysis of 8 studies containing 854 cases indicated that Chinese herbal bath therapy group was superior to the control group in terms SCORAD index [(95%(-0.99, -0.55), *P* < 0.00001)] and recurrence rate [(95% CI (0.10, 0.59), *P* = 0.0002] among other outcomes and high safety profile [15, 16].

**Fig. 1:**
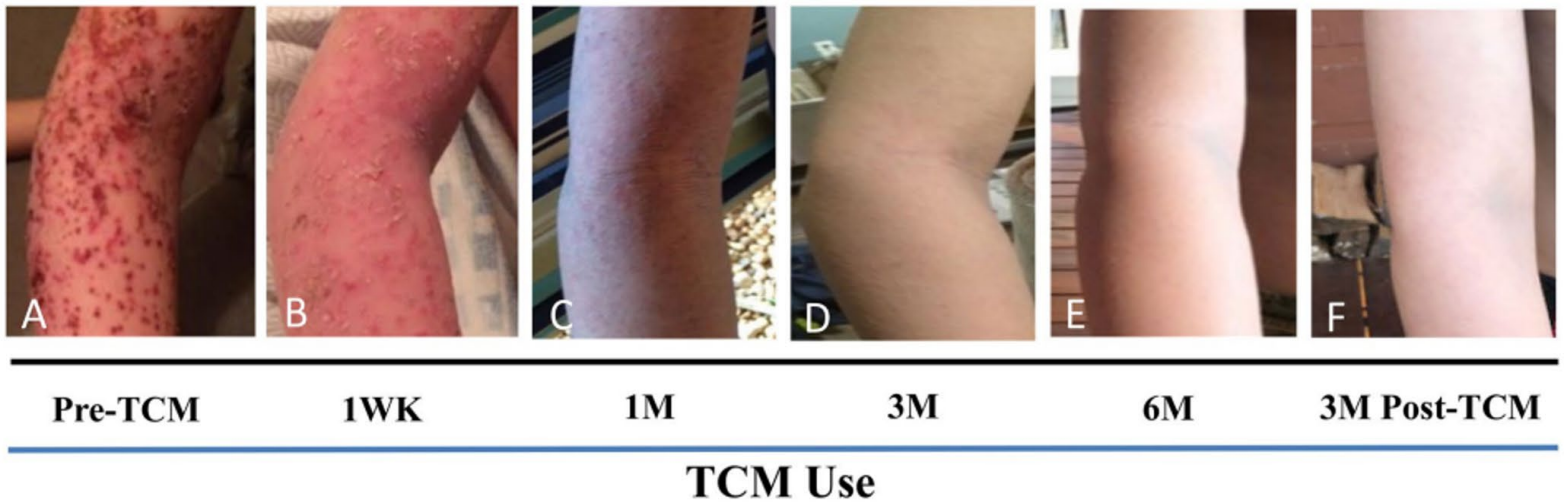
The effect of TCM on pediatric eczema. Progress from a 7 year old severe eczema pediatric patient during treatment. WK = week, M = month. Figure from Ref: Uzun et al., 2021

Case reports of children prescribed combined TCM therapy, consisting of *Phellodendron chinensis* herbal bath additive with additional herbal creams and internal tea, demonstrated good tolerability and clinical outcomes [17]. This clinical study completed by Thanik et al. consisted of 14 patients with moderate to severe AD whereby median age of study participants was 5.4 years, and the median duration of disease was 40 months. The TCM treatment, which lasted a minimum of three months, included a combination of herbal bath additive, herbal cream, and internal tea. The results showed a significant improvement, with the median scoring atopic dermatitis (SCORAD) score decreasing from 89 to 11 (*p* < 0.001), and the median dermatology life quality index (DLQI) score decreasing from 17 to 1 (*p* < 0.0001), indicating a significant change in baselines. Additionally, some patients reported stopping the use of antihistamines, topical steroids, and oral steroids, without flare, indicating a potential steroid sparing effect. The treatment was well tolerated, with the only inconvenience being skin discoloration after applying the creams due to *Indigo naturalis* herb. This coincided with the in vitro and in vivo, safety and Th2 axis modification demonstrated by reduced IL-4 production [17]. Further, in a case of a 7 year old male with refractory disease and corticosteroid withdrawal syndrome, the combined oral and topical TCM rapidly improved clinical measures of disease (Fig. 1) [18]. This included improved sleep quality, resolution of itching, oozing, and erythema, and reduced IgE and absolute eosinophil counts [18]. Notably, TCM was tapered without complications and maintained 3 months of the observation period after cessation [18].

Similarly, a study by Liu et al. of a twelve-week treatment of 275 patients aged 5–25 years with moderate-to-severe AD showed that in Chinese herbal-medicine treated groups mean SCORAD significantly decreased and quality of life significantly increased [19]. Furthermore, Chen et al. [20]explored the potential of TCM as a treatment option that can lower the use of corticosteroids for pediatrics with eczema. The study focused on children under the age of 12, and the use of corticosteroid after treatment was compared with TCM nonusers [20]. After the one year follow up, the use of TCM significantly reduced exposure to corticosteroids. The results showed that the corticosteroid use of the children who used a TCM regime was reduced by 42%, while it increased 34.5% in TCM nonusers and TCM users took steroids for a shorter time and had a decrease in doctor visits where the steroids were prescribed (*p* < 0.001) [20]. Therefore, lower usage of corticosteroids is possible in children after starting TCM treatment. Authors elaborated on possible mechanisms for their combined formula, including the antioxidant and anti-inflammatory effects of XFS through immunomodulation of Th1/Th2 in vivo and inhibition of IgE-dependent histamine release from mast cells [20]. Additionally, *Glycyrrhiza uralensis* and *Forsythia suspensa* demonstrated anti-inflammatory and anti-oxidation effect in vitro [20]. Furthermore, *Forsythia suspensa*,* Dictamnus dasycarpus* and *Paeonia suffruticosa* suppress mast cell degranulation and histamine release both in vivo and in vitro [20]. Therefore, alterations to the Th2-mediated pathway are largely the mechanisms of action of these formulas.

Srivastava et al. [21]completed a study to evaluate efficacy and safety of TCM to treat eczema. In cohort #1 (*n* = 10) patients aged 6–48 months had previously used topical steroids for at least 3 months with no improvement. The TCM regimen consisted of external herbal bath, cream and oral tea. In just one month, Cohort #1 had a decrease in SCORAD score and steroid use close to zero after 6 months (*p* < 0.001) [21]. Animal models confirmed reductions in SCORAD score, scratching, and skin eosinophil counts, with in vitro mechanisms highlighting dose-dependent reductions in IgE, TNF-α, and exotoxin production. This study confirmed that TCM is effective and safe in children with steroid dependent-eczema.

### Pediatric Urticaria

Uticaria, also known as hives, affects about 20% of people at some time in their lives, and this can be acute or chronic. The cause of these raised itchy rashes can be due to allergens, physical triggers, or underlying health conditions. Papular urticaria, which commonly affects children, is a hypersensitivity specifically to arthropod bites, most commonly mosquitoes, fleas, and bedbugs. In the case of pediatric allergic disease, hives develop in response to an allergen, whereby the release of histamine causes capillary leakage and results in fluid accumulation that causes inflammation and a rash. Prevalence peaks at 2 years old and tolerance is typically developed around 10 years old. Urticaria can either be acute spontaneous, chronic spontaneous or chronic inducible. Acute lasts less than 6 weeks, often resolving on its own, while chronic persists for over 6 weeks, sometimes recurring or lasting for years. Chronic urticaria has no identifiable cause, but it may be related to autoimmune disorders or underlying medical conditions. Current treatment for acute urticaria includes antihistamines, soothing anti-itch creams, and short-term use of topical steroids. In the case of chronic urticaria, regular antihistamine usage or Omalizumab for those under 12 years old are used [22–24]. As described, there is a need for safer and sustainable therapeutics.

Chronic spontaneous urticaria (CSU) is characterized by chronic or recurrent papules due to the response to the saliva and associated proteins released by the arthropod. These wheals and/or angioedema are debilitating, with significant impacts on sleep, school performance, and quality of life [22]. Recurrence typically lessens when adolescence and adulthood are reached. Histopathology samples demonstrated that 86% of patients present with eosinophils and IL-4 secreting CD4 + cells and lower IL-10 [25]. One review highlighted the immunomodulatory mechanisms of aged garlic extract, curcumin, zinc, balneotherapy (immersion in a thermal mineral water bath), quercetin, and *Rhododendron tomentosum*, however none of these studies included children [25].

### Alternative Medicine for Pediatric Urticaria

Few authors have suggested acupuncture is beneficial for up to 90% of chronic urticaria cases [26]. A double blind study of acupuncture for chronic urticaria treatment demonstrated a 25% reduction in episodes compared to placebo and reduced duration of episodes [27]. However, this study was performed in adults around 30 years of age and therefore would need further evidence for success in children [27]. This was also seen in other studies, with improved quality of life in adults, including in conjunction with Omalizumab [28, 29].

**Fig. 2:**
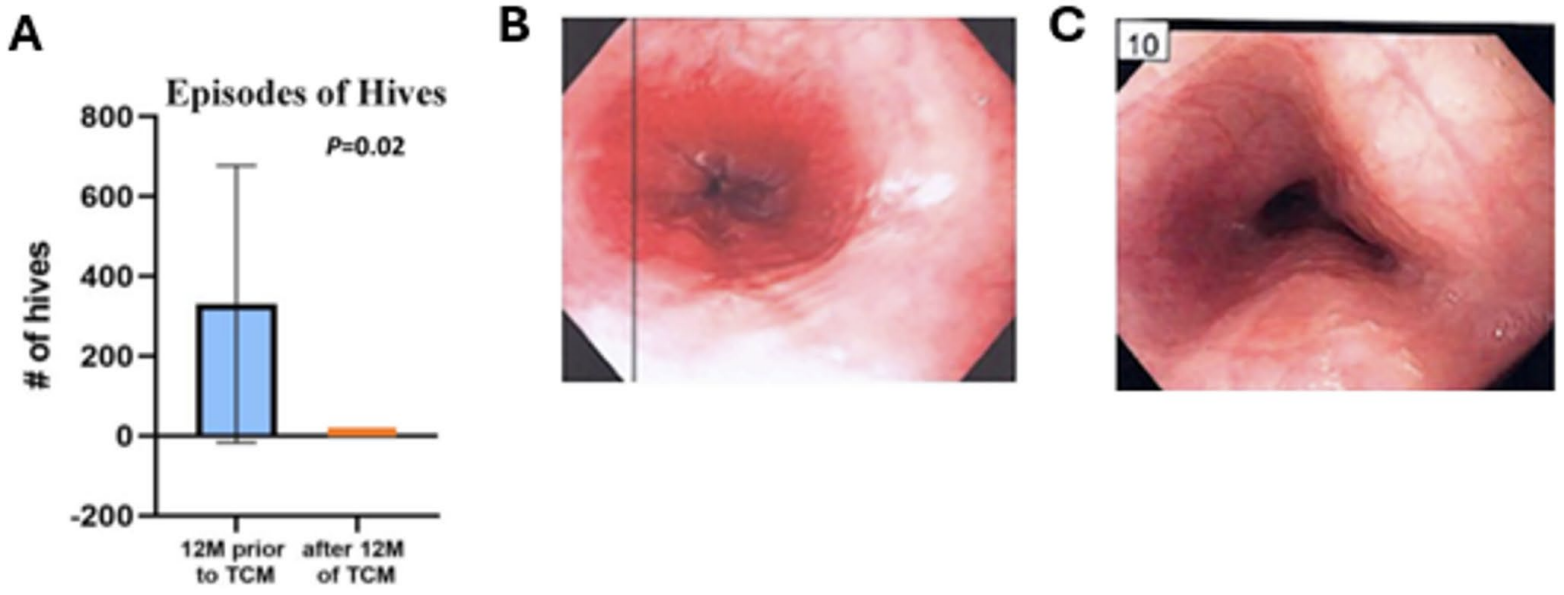
The effect of TCM on urticaria and EoE. (**A**) Effect of internal and external TCM treatments on pediatric chronic urticaria, including herbal baths and creams, and teas. Incidence of hives with TCM therapy. “Prior to TCM” group reflects a mean total of all three patients, calculated 1 year prior to initiating TCM therapy. “After TCM” reflects the mean total events occurring 1 year after starting TCM. Figure from Ref: Fan et al., 2022. (**B**) Effect of digestive tea formula on 11-year old boy with eosinophilc esophagitis, initial endoscopy demonstrating significant inflammation. (**C**) Follow-up endoscopy demonstrating significant improvement. Figures from Ref: Soffer et al., 2020. https://medicine.yale.edu/yjbm/openaccess/

Fan et al. demonstrated successful management of chronic urticaria and food allergies in a small retrospective case study of patients ranging from 20 months − 12-years old [30]. Patients received internal and external TCM treatments, including herbal baths and creams to reduce food reactions, including frequent hives. By 9 months, all patients had complete remission of atopic symptoms associated with reduced food-specific and total Ig levels, including hives (*p* = 0.02; Fig. 2A), epinephrine doses (*p* = 0.004), and allergy-related ER visits (*p* = 0.005) [30]. Therefore, TCM therapy may serve as a treatment option for multiple food sensitivities and chronic urticaria (Table 1).

## Pediatric Eosinophilic Esophagitis (EoE)

Eosinophilic esophagitis is a chronic inflammatory upper gastrointestinal (GI) disorder that is characterized by esophageal dysfunction, including inflammation of the esophagus, abdominal pain, dysphagia, and food impaction. Food allergens primarily trigger the stimulation of inflammatory cells through the impacted esophageal barrier. Histologically, EoE is characterized as greater than 14 eosinophils on a biopsy, in addition to symptomology and pathology. This disorder, which is rapidly increasing in prevalence (1 case per 1000 people), has very limited effective conventional treatments [31]. Currently, common treatment options include dietary elimination, which is difficult and cumbersome, proton pump inhibitors, which has a varying response rate in children, topical steroids, the viscous budesonide, and Dupilumab. Budesonide, a systemic steroid medication, is the first-line recommended drug for EoE, however its efficacy remains controversial [32]. While systemic steroids are easily administered and have a high and rapid response, adverse long term effects of steroids and poor solubility are evident. Another steroid, the swallowed fluticasone, is FDA-approved for asthma, but is prescribed off-label for EoE. Additionally, discontinuance of systemic steroids results in high rates of recurrence. Ranitidine (Zantac), a histamine blocker that treats GI reflux and heart burn, was withdrawn by the FDA due to increased cancer risk. Dupilumab, the only FDA-approved treatment for EoE, is a monoclonal antibody that blocks Th2 cytokines IL-4 and IL-13. Dupilumab, however, has negative side effects and relapses following cessation of therapy. As a chronic disease, management requires a therapy that is long-term and multidisciplinary. Therefore, the use of less conventional therapeutics has been investigated (Table 1).

### Complementary Medicine for EoE

Cross-sectional surveys in Australia have demonstrated 75% of children utilize complementary medicine when self-used by their carers [33]. This had been previously established and largely attributed to the unmet needs met by use of conventional care [34]. Additionally, Healthline suggests that for alleviation of EoE symptoms, one can try herbal remedies like licorice, chamomile, or acupuncture as these have demonstrated effects on gastroesophageal reflux disease (GERD), however, this has only been demonstrated in adults [35–37]. In fact, one patient has self-reported usage of acupuncture and its success in mitigating her symptoms of EoE [38].

### Digestive Tea Formula for EoE

TCM practices have utilized Digestive Tea Formula (DTF) to alleviate GI symptoms. In a case report, an 11 year old that was diagnosed with EoE and presented with refractory disease, was initiated on TCM therapy. This therapy, including Digestive Tea, topicals, acupressure, and acupuncture, resulted in disease resolution (Fig. 2B) [39]. The identification of the major active compounds resulted in the investigation of 7,4 dihydroxy flavone (DHF), a flavonoid purified from *Glycyrrhize uralensis*. This compound has demonstrated inhibition of the allergic response in vitro and in vivo, including eotaxin, IgE, and Th2 cytokines, including TNF-alpha, IL-6, and IL-8 (*p* < 0.001) [39, 41]. This poses DHF as a therapeutic candidate for EoE.

## Pediatric Food Allergy

Food allergies (FA) affect approximately 8% of children in the United States and remain a growing public health concern [42]. Currently, there is no cure, and treatment strategies mainly focus on avoidance and symptom management [43–45]. Monoclonal antibodies such as omalizumab and oral immunotherapy (OIT) show promise but have notable limitations [46]. Omalizumab, an anti-IgE antibody, binds to the Cε3 domain of free IgE, preventing its interaction with FcεRI on mast cells and basophils. Yet, in clinical trials, omalizumab’s effects have been variable including complete treatment failures and allergens still need to be avoided [47]. OIT, such as Palforzia for peanut allergies, requires daily administration to maintain desensitization and loss of desensitization can occur over time [48], and not recommended for patients with severe reactions [49]. These challenges highlight the need for safer and more effective long term effect for FA.

### Development of Food Allergy Herbal Formula-2 (FAHF-2) and Derivatives

**Fig. 3:**
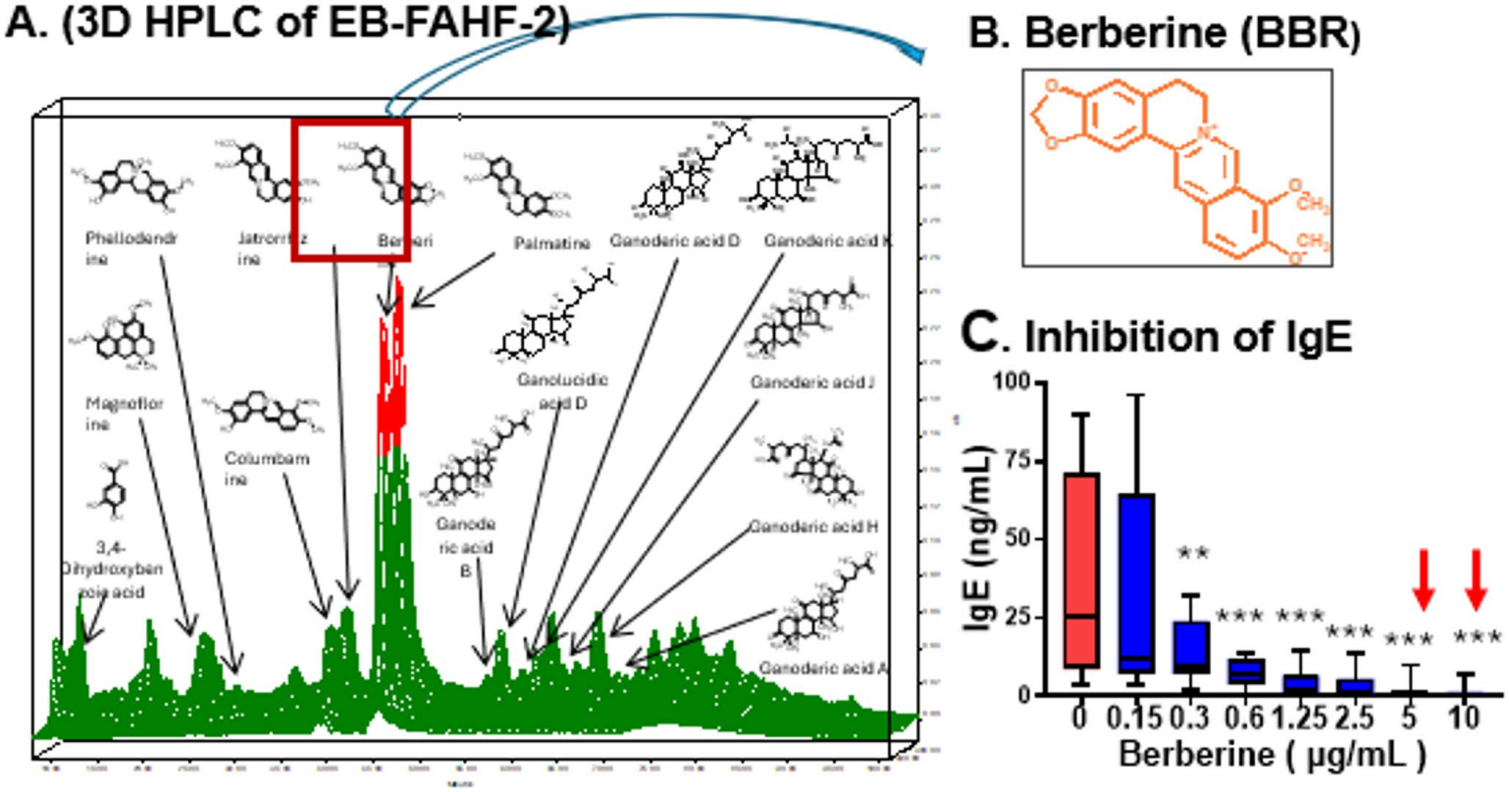
Identification berberine as anti-IgE food allergy treatment. (**A**) 3 Dimensional HPLC fingerprint of E-B-FAHF-2 showing berberine is a major component. (**B**) Berberine structure. (**C**) BBR dose-dependent inhibition of IgE production by peripheral blood mononuclear cells from patients with a variety of food allergies. **P < 0.01; ***P < 0.001 (*n* = 7–10). Ref: Yang et al., 2014

FAHF-2 was derived from a TCM antiparasitic formula *Wu Mei Wan* [50], since food allergy and intestinal parasite infection share a Th2 dominant immune response, including elevation of IgE and activation of mast cells and eosinophils. Advanced extraction methods, such as butanol and ethyl acetate extraction, were used to enhance the bioactivity of key compounds, leading to the development of enhanced formulations such as butanol-extracted FAHF-2 (BF-2) and a further refined version, ethyl acetate and butanol-extracted FAHF-2 (EBF-2). FAHF-2 showed significant immunomodulatory effects in preclinical studies, supporting its potential application in FA treatment. In murine models of peanut allergy, FAHF-2 reduced anaphylactic reactions, lowered histamine levels, and decreased basophil and mast cell populations and peanut-specific IgE without negative effect on IgG2a [51–53]. The combination of BF-2 and OIT reduced adverse events and promoted cytokine shifts favoring a Th1 and T regulatory phenotype, with altered DNA methylation patterns in immune-related genes such as significant enhancement of methylation levels at IL-4 promoter, as one of the mechanisms underlying BF-2 suppression of IL-4 [54]. EBF-2 demonstrated long-term efficacy by reducing IgE levels and protecting against anaphylaxis for up to 52 weeks post-treatment. Flow cytometry analysis showed a decrease in IgE+/CD138 + plasma cells [55]. Berberine (BBR) has been identified as a key IgE inhibitory compound from FAHF-2/EBF2 and inhibited IgE production (90–100%) by peripheral blood mononuclear cells from food allergic patient at very low doses without cytotoxicity [56] (Fig. 3). BBR also inhibited IgE production by a human IgE-producing plasma cell line (U266 cells) and revealed downregulation of transcription factors such as XBP1, BLIMP1, and STAT6, critical for plasma cell activation and IgE production [55]. *Angelica sinensis* (AS), a component of FAHF-2, was shown to enhance the uptake of berberine (BBR) by CACO-2 cells. A combined treatment of BBR, AS, and boiled peanut oral immunotherapy (BNP) led to long-term peanut tolerance in allergic mice, with reductions in IgE levels, allergic symptoms, plasma histamine, body temperature, and the number of IgE⁺ B cells (*p* < 0.001 vs. Sham). The BNP treatment also altered the gut microbiota, with significant changes in the Firmicutes/Bacteroidetes ratio across groups [57]. The development of nano-formulation encapsulated BBR has overcome BBR’s inherently poor bioavailability, a barrier to its use (Li et al. US patent has been issued [58]).

### Clinical Trials of FAHF-2 and EBF-2

The initial Phase I trial [59] was a 7-day randomized, double-blind, placebo-controlled, dose-escalation trial (median age: 16 years, range: 12–33 years). The primary objective evaluated the safety and tolerability of FAHF-2 in 18 patients (12 FAHF-2 and 6 placebo). The secondary objective was to evaluate the immunomodulatory effects of FAHF-2. The study demonstrated a favorable safety profile, with no grade 3 adverse events reported, showed reduction of IL-5 production following 7-day treatment compared with the placebo group. In addition, PBMCs collected from baseline were cultured with FAHF-2 in vitro showed a significant reduction in IL-5 (*p* = 0.024) and an increase in IFN-γ (*p* = 0.019) and IL-10 (*p* = 0.01), suggesting a shift from a Th2 to a Th1 and T regulatory response.

In the extended Phase I study [60]14 of 18 participants (median age: 16 years, range: 12–27 years) completed 6 months of treatment with FAHF-2, demonstrating its long-term safety. A significant reduction in basophil activation was observed after 4 months and 6 months of treatment (compared to baseline (*p* < 0.05, and *p* < 0.01 respectively). In the initial acute Phase I study, several participants reported that the high dose (12 tablets thrice daily) posed a significant burden and deterred compliance, leading to a reduction in the number of tablets administered.

The Phase II study [61] of FAHF-2 was a double blind, randomized, placebo-controlled study, that included 68 subjects (median age: 16 years, range: 12–45). This study confirmed the safety of FAHF-2 but failed to demonstrate significant differences between the active and placebo groups in the primary endpoint i.e., the percentage of patients who could eat 2 g of protein or tolerate at least four times more allergen after treatment compared to baseline. While 86.8% of participants completed the study, 44% had less than 80% adherence for at least 2 months of the 6-month study, including one-third during the last 4–6 months, possibly causing suboptimal dosing for the treatment. Interestingly, experiments using PBMCs from the same study participants stimulated with food challenges in the presence or absence of FAHF-2 in vitro significantly reduced IL-5 (*p* = 0.01) and increasing IL-10 levels (*p* = 0.003), along with an increase in CD4 + CD25 + FoxP3 + regulatory T cells (*p* = 0.045), with no cytotoxicity compared to PBMCs stimulated with food allergies without FAHF-2, demonstrating if the cells exposed to sufficient active compounds, a potential switching from Th2 to Treg may occur. An additional multicenter, double-blind, placebo-controlled Phase II trial [62] investigated the combination of an enhanced butanol purified FAHF-2 (E-B-FAHF-2) with omalizumab and multi-OIT (EOIT). Of the 33 participants enrolled, only 18 completed the study (median age: 11.7 years; range: 6–40), but all data are analyzed. E-B-FAHF-2 was well tolerated. At 26 months, 63.6% of participants were desensitized to 4444 mg of protein for each allergen, and by 29 months, 24.2% achieved remission, with no differences between treatment groups. Among the allergens tested, milk skin prick tests (SPT) showed a significant reduction in EOIT (*n* = 9) but not OIT groups (*n* = 8) at immediate and 3 months post therapy (*p* < 0.001). It also showed a greater reduction of peanut and walnut skin prick tests for 3 months post-therapy in EOIT (*p* < 0.01 − 0.001), but not OIT group. However, this is a small sample size study, further research is needed before drawing clear conclusions.

Taken together, FAHF-2 and its refined version showed a high safety profile, beneficial immunomodulatory effect in vivo in early-stage clinical studies and in vitro, i.e. reduction of Th2 cytokines and basophil activation, increase IFN-γ, Il-10, and Tregs as well as skin prick tests (Table 1). However, the clinical effect remains to be established. The pronounce immunomodulatory effect suggests that if a sufficient amount and time of active compounds were able to enter the circulation, it may switch allergic prone to a tolerogenic immune response. Future clinical study needs to improve drop-out rate by increasing the convenience and potency including bioavailability production via convenient oral administration.

### Small Molecule Compounds Arctigenin as Novel Therapeutic Candidate

**Fig. 4:**
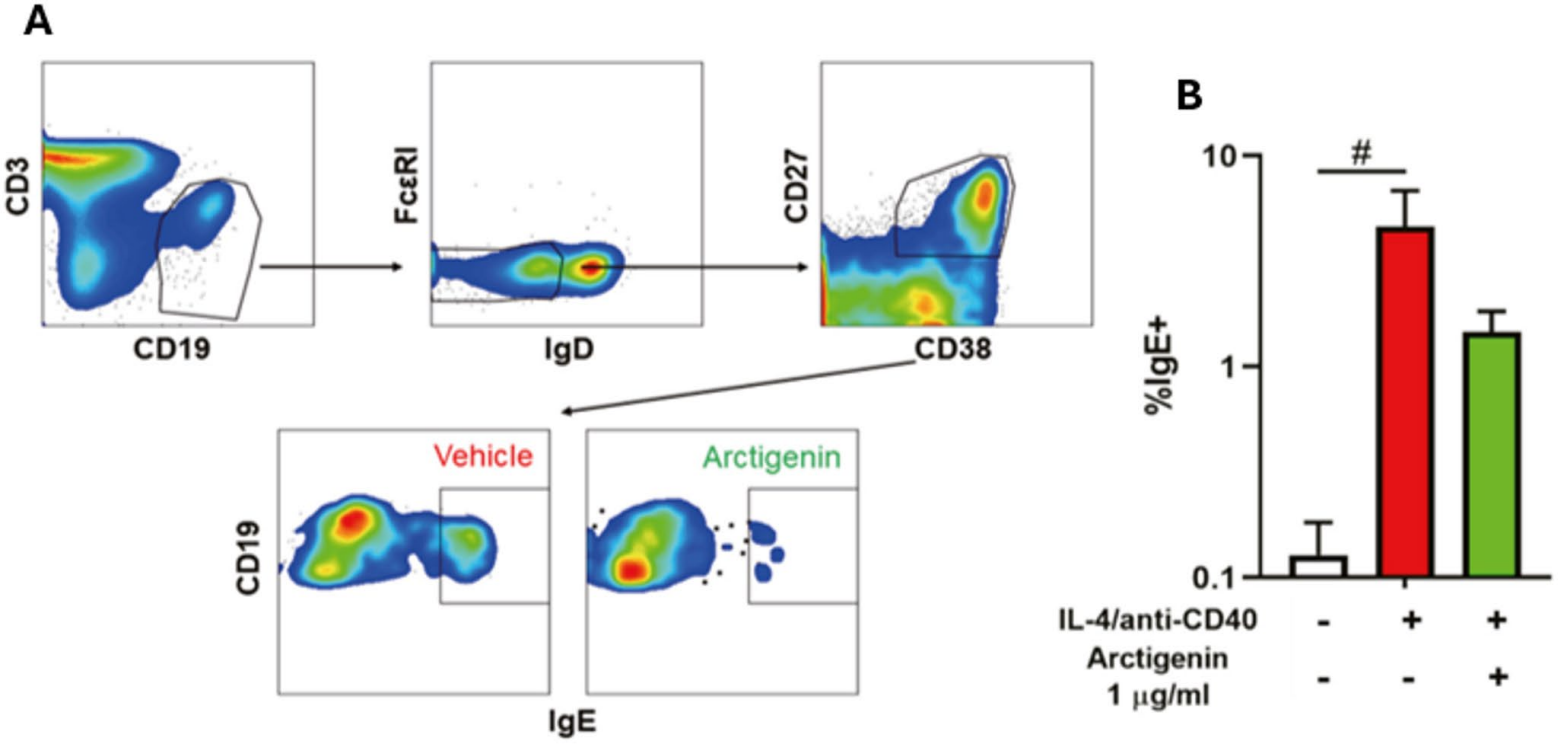
The effect of arctigenin on food allergy. (**A**) Inhibitory effect of arctigenin on IgE production by human PBMCs gating strategy for IgE + B cells and (**B**) frequency of IgE + B cells among PBMCs stimulated with IL-4 and anti‐CD40 in the presence (green) or absence (red) of arctigenin at 1 µg/ml after 7 days of culture. #*p* < 0.05 vs. unstimulated (*n* = 6). Figures from Ref: Cao et al., 2022

Arctigenin, isolated from *Arctium lappa*, has shown potential as a therapeutic candidate for food allergies. Cao et al. [63]analyzed PBMCs from 26 food-allergic patients and found that arctigenin significantly reduced IgE secretion in IL-4 and anti-CD40-stimulated PBMC cultures in a dose-dependent, non-cytotoxic manner (Fig. 4). Transcriptomic analysis revealed 479 differentially expressed genes, with downregulation of genes involved in B cell proliferation and survival and upregulation of BCL6, an IgE suppressor [64]. Arctigenin also modulated T cell responses, reducing IL-5 (*p* < 0.0001) and IL-13 (*p* < 0.001) levels after αCD3/CD28 bead stimulation.

## Allergic Rhinitis

Allergic rhinitis (AR) is one of the most common inflammatory disorders of upper respiratory tract in children. The symptoms of AR include nasal congestion, runny nose, itching eyes, sneezing and throat irritation. The prevalence of AR has increased significantly in recent decades. More than 400 million people suffer from AR globally [65]. Up to 40% of children in US suffered from AR [66]. Conventional treatment options include oral antihistamines such as loratadine, cetirizine; using intranasal corticosteroids inhaler (fluticasone, mometasone). However, the antihistamines medicines can cause drowsiness and cardiac toxicity. Long-term using corticosteroids might lead to upper respiratory tract infections, coughing or nose bleeding. TCM has been used to manage AR in children for a long time. According to the TCM theory, AR is due to deficiencies in the lung, spleen, and kidney systems. The weakened or imbalanced Qi in children in digestion system making children more susceptible to allergens. TCM treatments are used to restore balance and strengthen the immune system. Herbal medicine, acupuncture, and pediatric massage methods have been used clinically to improve the symptoms and quality of life for children (Table 2).

**Table 2:** Summary of effects of TCM on pediatric allergic rhinitis and asthma

Treatment	Clinical Trials	Mechanism of Action	Ref
*Pediatric Allergic Rhinitis*			
**Yu Ping Feng San** (Jade Wind-Barrier) Astragali Radix (Huangqi), Atractylodis Macrocephalae Rhizoma (Baizhu), and Saposhnikoviae Radix (Fangfeng)	Sample size *n* = 50 Loratadine; *n* = 51 Jade Wind-Barrier powder + Loratadine Age range: 3 ~ 12 years Length of study: 28 days	Improved symptom scores	[69]
**IMMBO** Cedrus deodara, Curcuma longa, Cypus rotundus, Emblica officinalis, Emblica ribes, Holarrhena antidysentrica, Picrorrhiza kurroa, Berberis aristata, Piper longum, Piper longum (Root), Piper nigrum, Plumbago zeylanica, Saussurea lappa, Terminallia belerica, Terminallia chebula, Zingiber officinalis, Boerhavia diffusa, Operculina terpathum	Age range: 4 ~ 60 (Subgroup 4 ~ 12) Length of study: 28 days	Reduced IgE Improved symptom scores	[70]
**Acupoint** Herba Ephedrae, Herba Asari, Cortex Cinnamomi, Semen Sinapis Albae, Radix Scutellariae, and Borneolum Syntheticum	Sample size: *n* = 60 Montelukast; *n* = 60 Acupoint + Placebo; *n* = 60 Acupoint + Montelukast Age range: <16 years Length of study: 12 weeks	Improved symptom scores	[72]
**Moxibustion plus loratadine**	Sample size: *n* = 40 Loratadine; *n* = 40 Moxibustion + Loratadine	Improved symptom scores	[73]
*Pediatric Asthma*			
**XiaoQingLongTang** (XQLT) Herbal formula: Ephedrae herba (Ma-Huang), Cinnamomi ramulus (Gui-Zhi, ), Paeoniae radix alba (Bai-Shao), Herba cum Radix Asari (Xi-Xin), Zingiberis Rhizoma (Gan-Jiang, ), Pinelliae rhizoma (fa-Ban-Xia), Schisandrae chinensis fructus (Wu-Wei-Zi), Glycyrrhizae Radix et rhizoma (Gan-Cao)	Sample size *n* = 40 Budesonide; *n* = 40 XQLT Length of study: 7 days Age range: 5 ~ 13 years old	Improved FEV1 Improved symptom score	[77]
**JianXianQingLongTang** Herbal formula: Ephedrae herba (Ma-Huang), Cinnamomi ramulus (Gui-Zhi, ), Paeoniae radix alba (Bai-Shao), Herba cum Radix Asari (Xi-Xin), Zingiberis Rhizoma (Gan-Jiang, ), Pinelliae rhizoma (fa-Ban-Xia), Schisandrae chinensis fructus (Wu-Wei-Zi), Glycyrrhizae Radix et rhizoma (Gan-Cao). Poria (Fu-Ling), Semen Armeniacae Amarum (Xing-Ren), Pheretima (Di-Long), and Citri Reticulatae Pericarpium (Chen-Pi)	Cough variant asthma Sample size: *n* = 61 Fluticasone propionate inhalation; *n* = 61 JXQL + Fluticasone Length of study: 8 weeks Age range: 3 ~ 14	Improved FEV1 Improved symptom score	[78]
**Zhen-Wu-Tang** Herbal formula: Radix Aconiti Lateralis Preparata (Fu-zi); Poria cocos (Fu-ling); Rhizoma Atractylodis Macrocephalae (Bai-zhu); Rhizoma Zingiberis Recens (Sheng-jiang); Paeoniae Radix Alba (Bai-shao)	Sample size *n* = 1 Age range: 38 years Length of study: 7 days	Improved symptom scores	[79]
**Shegan MaHuang decoction** Ephedra (Mahuang); Asarum (Xixin); Belamcanda (Shegan); Bitter Apricot Seed (Kuxingren); Pinellia (Qingbanxia); Schisandra (Wuweizi); Magnolia Flower (Xinyi); Licorice (Gancao);	Cough variant asthma Sample size *n* = 1 Age range: 6 years Length of study: 4 days	Improved symptom scores	[80]
**XieHuang Jiejing** (XHJJ) Ephedra (ma-huang), scorpio (quan-xie), Sophorae Flavescentis Radix (ku-sheng), Semen Armeniacae Amarum (xing-ren), Ginkgo biloba Linn (bai-guo), and Radix Glycyrrhizae (gan-cao)	Cough variant asthma Sample size: *n* = 60 placebo; *n* = 120 XHJJ granules Age range: 4 ~ 7 years Length of study: 7 days	N/A	[81]
**Moxibustion plus TCM** Ephedrae herba (Ma-Huang), Cinnamomi ramulus (Gui-Zhi, ), Paeoniae radix alba (Bai-Shao), Herba cum Radix Asari (Xi-Xin), Zingiberis Rhizoma (Gan-Jiang, ), Pinelliae rhizoma (fa-Ban-Xia), Schisandrae chinensis fructus (Wu-Wei-Zi), Glycyrrhizae Radix et rhizoma (Gan-Cao)	Cough variant asthma Sample size: *n* = 45 montelukast tablets; *n* = 45 herb-partitioned moxibustion Age range: 3 ~ 12 years Length of study: 6 weeks	Reduced IgE Improved FEV1 Improved symptom scores	[94]
**Acupuncture**	Chronic persistent asthma Sample size: *n* = 60 fluticasone propionate aerosol, *n* = 60 acupoint thread-embedding	Reduced IgE Improved FEV1 Improved symptom scores	[99]
**Acu-TENS**	Sample size: *n* = 32 breathing restraining, *n* = 32 breathing restraining + Acu-TENS Age range: 12–16 years old	Reduced IgE Improved FEV1 Improved symptom scores	[101]

*Abbreviations*: FEV1 (Forced Expiratory Volume in 1 s)

### Herbal Medicine for Allergic Rhinitis

Traditional Chinese herbal formula has shown strong potential in managing the symptoms of allergic rhinitis in children. A meta-analysis study, which incorporated 23 randomized controlled clinical investigations, showed that the applying Traditional herbal medicine alone or combined with conventional treatment significantly improved the therapeutic effect and alleviated the nasal itching symptoms in AR children compared with placebo or conventional treatments [67]. The TCM treatment also regulated the immune system by increasing the IL-10 level, reducing the serum IgE, IL-4, and IL33 levels [67]. 

A meta-data analysis of TCM formula, Cang-Er-Zi-San (CEZS), for pediatric AR included 15 RCTs with 1361 children aged 0 to 18 years old included (692 in the CEZS group and 669 in the control group). Results showed that CEZS significantly improved the symptoms of AR compared with other therapies. The overall effective rate was significantly higher than control group (*p* < 0.00001). The nasal congestion and runny nose relief time was significantly shortened by CEZS compared with other therapies (*p* < 0.00001). Further pharmacological research studies suggested that CEZS has anti-inflammatory and antibacterial properties. It inhibited the nasal bacterial growth, reduced the swelling, constricted the nasal mucosal blood vessels, and alleviated the allergic symptoms. The CEZS treated group showed lower recurrence rates, which will reduce the risk of further developed asthma conditions. CEZS also showed a strong safety margin. In the 15 published studies, only three adverse events were reported [68]. 

Another commonly used TCM formula is Yu Ping Feng San (also called Jade Windscreen Powder). In 2021, Dr. Zhou published the results of applying Jade Wind-Barrier powder in 101 AR children. 50 children treated with JWBP combined with Loratadine showed significant higher response rate (96.08%), shorter symptom disappearance time, lower symptom scores, lower levels of IL-13, IL-4 and TNF-α, higher CD4 + CD25+, CD19 + and CD8 + cells compared with 50 children treated with Loratadine only. The JWBP combined treated children also showed a significantly lower recurrence rate (3.92%) compared with conventional treatment (26%) [69]. 

Herbal medicine was also widely used in India as the treatment for AR. A herbo-mineral formulation (IMMBO) consisting of 18 herbs was investigated in a randomized, comparative, clinical trial including 250 AR patients age from 4 ~ 60. Patients received daily treatment of either IMMBO formula or the combination of levocetirizine and montelukast for 28 days. Results showed that both IMMBO treated and conventional medicine treated groups showed significant improvement in nasal symptom scores. The IMMBO formula treated group showed higher reduction nasal symptom scores compared with levocetirizine and montelukast treated group. The subgroup analysis showed that pediatric patients (age from 4 to 12 years) showed a similar trend as the overall groups [70]. 

### Acupuncture or Acupoint Pressure Method for Allergic Rhinitis

Acupuncture has been widely used as a commentary and alternative treatment for AR. As non-pharmacological therapy, acupuncture as the treatment for AR in children showed a very safe record in reducing the inflammation and alleviating the nasal congestion. Very few cases of side-effects of acupuncture treatment were reported. Even these reported cases are only related to slight pain or minor bruise and were recovered very soon. In a comprehensive meta-analysis study of 13 RCT and 1186 AR patients, data showed that acupuncture combined with conventional medication significantly increased the effectiveness rate compared with medication only group (RR = 1.29, 95% CI = 1.17 to 1.42, *p* < 0.00001) and the recurrence rate was also significantly reduced [71]. In a randomized, placebo-controlled and single-blind trial, 180 children with perennial AR were separated into 3 groups and treated with Chinese medicine acupoint application only, or Montelukast only, or combined treatment. After 12 weeks treatments, the TCM acupoint application only or combined treatment group showed significant higher evaluation scores of different AR symptoms (nasal congestion, sneezing, sleep problem, ang rhinitis symptom) compared with Montelukast only group (*p* < 0.05). And the Th2% was significantly reduced in acupoint treated groups [72]. 

### Moxibustion for Allergic Rhinitis

Moxibustion method combined with conventional treatment was also investigated for its effect on allergic rhinitis in children. A randomized, controlled clinical trial of 80 children with AR were separated into two groups. Control group was treated with loratadine tablets only while the observation group was treated with mild moxibustion and loratadine tablets. Results showed that the nasal symptom scores, the serum eosinophils counts were significantly decreased in both groups. Interestingly, the effective rate value was higher in the treatment combined with moxibustion compared with conventional treatment only and the nasal symptom scores were lower in moxibustion treated group [73]. 

### Pediatric Tuina for Allergic Rhinitis

Pediatric Tuina is a special therapeutic massage method commonly utilized in China for infants and children. The techniques include pressing, pinching, or pushing on specific acupoints or part of a child’s body. TCM theory believes that this massage technique could regulate the imbalanced Qi strengthen the blood circulation and improve the function of target organs. Pediatric tuina has long been used as the treatment on AR in children. A meta-analysis reported the effectiveness of pediatric tuina on AR in12 RCTs including 718 children. The clinical effectiveness was shown significantly higher in pediatric tuina treated children with AR (RR = 1.16, 95% CI:1.08–1.25, *p* < 0.01). The symptoms of nasal congestion, runny nose, sneezing, and turbinate swelling were also significantly improved when compared with conventional treatment only (*p* < 0.01) [74]. 

## Pediatric Asthma

Allergic asthma is a chronic inflammatory disorder of the airway, which is characterized by bronchial hyperresponsiveness, airway remodeling, mucous secretion, inflammation, and immune dysregulation when triggered by allergens. Current treatment focuses on preventing the airway remodeling through inhaled corticosteroid or bronchodilators for quick relief. Montelukast, a leukotriene receptor antagonist, is used to block inflammatory pathways, and loratadine is used to suppress the histamine level to control allergic reactions. New treatment, such as Omalizumab, focus on reducing the IgE level in severe asthmatic patients [75]. However, recurrent asthma is still the challenge for all conventional treatments. TCM has been considered as an alternative or complementary treatment for allergic asthma (Table 2). Unlike western medicine, Herbal formulas, acupuncture, and moxibustion were used to relieve asthma symptoms. The data analysis of a population-based retrospective cohort study of 10,470 asthma patients showed that patients using TCM had significantly reduced medical utility of asthma admission, such as asthma emergencies and asthma admission, especially in patients who had TCM treatment > 60 days [76]. 

### TCM Herbal Formula on Asthma

TCM formula is the major therapeutic approach for treating allergic asthma. The most commonly used TCM formulas are Ding-Chuang-Tang, Ma-Xing-Gan-Shi-Tang, Ma-Huang-Tang, and Xiao-Qing-Long-Tang etc. Randomized controlled clinical trials of children with bronchial asthma [77] or cough variant asthma [78] were performed to investigate the effectiveness of Xiao-Qing-Long-Tang recently. It was found that Xiao-Qing-Long-Tang treatment combined with conventional western medicine significantly increased the effective rates compared with non-TCM treated group. After 1 week of treatment, the FEV1% value, the FEV1/FVC% and was significantly increased in TCM treated group vs. non-TCM treated group (*p* < 0.05) [77]. The modified Xiao-Qing-Long-Tang (with additional *Poria*,* Semen Armeniacae Amarum*,* Pheretima*, and *Citri Reticulatae Pericarpium*) also effectively improved the allergic asthma symptoms, reduced cough, and improved the FVC, PEF, FEV1/FVC values in child patients [78]. In 2024, Dr. Chan reported the successful treatment of a middle-aged female patient who suffered from severe asthma, struggled to take full breaths) by using Zhen-Wu-Tang formula. After one week of treatment, this patient experienced significant improvement with reduced mucus production and almost no breathing difficulties [79]. Other herbal formulas, such as Shegan Mahuang decoction for the clinical usage on variant asthma in children [80] and Xiehuangjiejing (XHJJ) granule for cough variant asthma [81] were also proposed in well-designed, double-blind, randomized, controlled trial for their anti-asthmatic functions. ASHMI, derived from TCM is composed of three herbs: Ling-Zhi (*Ganoderma lucidum*), Ku-Shen (*Sophora flavescens*), and Gan-Cao (*Glycyrrhiza uralensis*), showed clinical safety and efficacy in asthma in controlled studies [82, 83]. Substantial research has isolated and identified active compounds and revealed multi-levels of beneficial mechanisms, including direct modulating airway smooth muscle contraction (Yang) [84, 85], reduction of Th2 cytokines and increase Tregs (Chang data, Ying), reduced TNF-α (Zhen) and IgE production [86–91] These data will support the further understanding of TCM on allergic asthma and might bring new therapeutic options for allergic asthma.

### Moxibustion (Acupoints Therapy)

Acupoints therapy is another complementary treatment for asthma, which apply the moxibustion (burning dried mugwort), TCM, or acupuncture on specific points to improve the lung function, airway inflammation and asthma symptoms. Recently, a meta-analysis, which analyzed 37 randomized controlled trials of 2879 cases, showed that moxibustion significantly improved the lung function and asthma symptoms, reduced IgE levels when used alone or combined with conventional anti-asthmatic drugs. And the adverse events were mild [92]. A clinical trial data of 60 children with respiratory tract infection, which was published in 2021, showed that combination of moxibustion with pediatric tuina significantly increased the effectiveness rate. The symptoms scores and the total times of infections reduced significantly after the moxibustion treatment compared with pediatric tuina only [93]. Using modified Xiao-Qing-Long-Tang as the material for moxibustion on Geshu (BL17) and Danshu (BL19) on children with cough variant asthma for a total 6 weeks treatment significantly reduced the cough and increased the pulmonary airway function. The MEF75%, MEF50%, and MEF25% values were significantly higher than the control group treated with montelukast tablet only [94]. 

### Acupoint Therapy with TCM Formula

Acupoint therapy by applying herbal medicines directly on different acupuncture pints to facilitate the absorption of the medicine through skin has been wildly used to treat allergic asthma in China for a long time. The combination of acupoint therapy with conventional treatment can significantly improve the clinical effect in patients with severe bronchial asthma [95]. Meta-analysis study showed that acupoint therapy using herbal patching, such as white mustard seed, radix Kansui, and rhizome corydalis, on Sanfu Days showed important improvement in children with asthma. In children with asthma, the applying of these herbal patching on selected acupoints (Danzhong (CV17), Feishu (BL13), Geshu (BL17), and Tiantu (CV22)) showed significant improved asthmatic symptoms with very minimal side effect [96]. Application of acupoint massage with ear point of children with asthma also showed significantly improved the pulmonary function (FEV1), FVC, FEV1/FVC ratio. And the total clinical effective rate was higher in acupoint therapy treated groups compared with control group [97]. 

### Acupuncture on Allergic Asthma

Acupuncture is another option for preventing or treating allergic asthma. A systematic review and meta- analysis of randomized sham/placebo-controlled trials of 16 RCTs of adult asthma patients showed that acupuncture therapy could improve the lung function (FEV1%), quality of life, and reduced the asthma symptoms in asthmatic patients compared with sham/placebo treated patients [98]. However, only a few clinical trials were investigated in pediatric asthma patients in recent 5 years. In 2021, in a randomized, controlled trial of 120 children with persistent bronchial asthma, Dr. Yang et al. showed that the acupoint thread-embedding therapy combined with fluticasone propionate inhaler significantly improved the PEF, FEV1, and small airway function in children with chronic persistent bronchial asthma with significantly increased IgA level and reduced serum IgE level [99]. 

### New Method of Acu-TENS

The transcutaneous electrical nerve stimulation (TENS) unit has been created and used to stimulate acupoints (Acu-TENS) for decades. Recently, researchers and practitioners have been applying the Acu-TENS technique for treating asthma [100]. In a recent double-blind randomized clinical trial of 32 teenagers with asthma, Dr. Elnaggar showed that subjects treated with Acu-TENS at Dingchuan EX-B1 had significantly improved pulmonary function (FEV1%, FVC%, and FEV1/FVC) with reduced serum IgE level compared with control groups. The quality of life score in Acu-TENS group also increased significantly [101]. 

## Conclusion

The unmet need of therapeutic and preventative options for patients with chronic and quality of life-diminishing allergic diseases is of the utmost clinical importance. Current therapies come with their own side effects, are not reliable long-term options, and are expensive. The use of TCM, as monotherapy or integrative therapy, has provided potential for these patients, as evidenced by clinical trials, case studies, published abstracts, and meta-analyses. While it has been difficult to make definitive conclusions about TCM with their varied formulations and administration routes and the small sample size in human studies, studies are underway to identify the active compounds, and their mechanisms of action may provide potential for safe and effective natural products for pediatric allergies. Therefore, TCM, including herbs, acupuncture, and acupressure, have demonstrated much success in pediatric allergic diseases and should continue to be investigated.

## Data Availability

No datasets were generated or analysed during the current study.

## Key References

- Uzun S, Wang Z, McKnight TA, et al. Improvement of skin lesions in corticosteroid withdrawal-associated severe eczema by multicomponent traditional Chinese medicine therapy. Allergy Asthma Clin Immunol. 2021;17(1)10.1186/s13223-021-00555-. *Uzun et al. highlighted the use of TCM in pediatric eczema with significant clinical improvements that were sustainable.*
- Fan X, McKnight A, Neshiwat J, Park S, Chung D, Li X-M. Successful management of chronic urticaria and food allergies in a pediatric population using integrative traditional Chinese medicine therapy: a case series. Clin Mol Allergy. 2022;20(1):12. 10.1186/s12948-022-00175-y. *Fan et al. demonstrated high success of TCM in both immunological and clinical parameters of chronic urticaria in the pediatric population.*
- Soffer G, Kaman K, Li X-M. Successful Management of Eosinophilic Esophagitis Using Traditional Chinese Medicine: A Case Report. Yale J Biol Med. 2020;93(5):685-8. *This case report by Soffer et al. provided evidence for the use of TCM in pediatric eosinophilic esophagitis.*
- Yang N, Wang J, Liu C, et al. Berberine and limonin suppress IgE production by human B cells and peripheral blood mononuclear cells from food-allergic patients. Ann Allergy Asthma Immunol. Nov 2014;113(5):556–564.e4. 10.1016/j.anai.2014.07.021. *Yang et al. identified berberine in the suppression of IgE production, highlighting its role across various allergic diseases.*
- Cao M, Liu C, Srivastava KD, et al. Anti-IgE effect of small-molecule-compound arctigenin on food allergy in association with a distinct transcriptome profile. Clin Exp Allergy. Feb 2022;52(2):250–264. 10.1111/cea.14048. *Successful amelioration of IgE-associated outcomes was observed by Cao et al. in food allergy patient samples*.
